# Suffering and dying well: on the proper aim of palliative care

**DOI:** 10.1007/s11019-017-9764-3

**Published:** 2017-04-03

**Authors:** Govert den Hartogh

**Affiliations:** University of Amsterdam, Staten Bolwerk 16, 2011 ML Haarlem, The Netherlands

**Keywords:** Suffering, Pain, Existential suffering, Aim of palliative care, Dignity therapy, Dying well, Ars moriendi

## Abstract

In recent years a large empirical literature has appeared on suffering at the end of life. In this literature it is recognized that suffering has existential and social dimensions in addition to physical and psychological ones. The non-physical aspects of suffering, however, are still understood as pathological symptoms, to be reduced by therapeutical interventions as much as possible. But suffering itself and the negative emotional states it consists of are intentional states of mind which, as such, make cognitive claims: they are more or less appropriate responses to the actual circumstances of the patient. These circumstances often are such that it would rather be a pathological symptom not to be sad and not to suffer. Suffering, therefore, is sometimes and to some extent a condition to be respected. Although I do not dispute that the alleviation of suffering is the main aim of palliative care, in pursuing that aim we should acknowledge a constraint of realism.

## Outline

Suffering is an all-too-common experience in human life, in all its phases. But it looms particularly large at the end of life, for patients dying from a lethal illness like cancer, in particular during the days, weeks or months that follow when every treatment with the exception of palliative care has been stopped. Though occasionally it is one particular symptom that causes severe, even extreme suffering, in most cases patients have to cope with the combined effects of multiple symptoms. Their suffering may also be aggravated by events in their own life, the loss of a partner for example, or even by the memories of traumatizing events in their youth or of personal failings during the course of their life.

The alleviation of suffering is traditionally considered one of the main aims of medicine. But when Eric Cassell published his classical paper on the nature of suffering and the goals of medicine in 1982,^1^ medical literature had for a long time been almost silent about the phenomenon. In the wake of the palliative care movement a substantial number of empirical studies of suffering, in particular of suffering at the end of life, has been published since then, the flow of such papers accelerating in recent years. This paper is based on a sample of such studies that, because of their many similarities, may be considered representative of the literature. Almost all these studies refer to Cassell, and in particular to his ‘definition’ of suffering. So it seems fitting to start (“Cassell on suffering” section) by considering Cassell’s views in some detail. In this discussion I will make a number of conceptual points that I will rely on in later sections.

In a second step (“Empirical work on suffering” section) I will consider the new empirical work on suffering in the end-stage of life. All authors accept Cassell’s point that suffering is not merely a matter of pain and other physical symptoms, but in addition has psychological, existential and social dimensions. In other respects, however, as I will show, they only pay lip-service to Cassell’s authority.

The main criticism that I will make of this literature ("The constraint of realism" section) goes beyond Cassell’s own views, although it builds on them. Cassell conceives of suffering as ‘a specific state of severe distress related to the imminent, perceived or actual, threat to the integrity or existential continuity of the person’.^2^ This means that suffering is an attitude vis-à-vis a certain state of affairs, that is understood to endanger the person’s life, his identity or his social role. If that person perceives her situation as threatening, it is always a relevant question whether she has understood that situation correctly. Suffering is therefore not a brute mental fact that can simply be established by introspection, rather it is normally an occasion for reflection, for the person herself and her intimates. Is my situation really as threatening as I perceive it to be, and how can I best cope with that threat? I do not deny that professional care-givers could play a modest role in this dialogue, providing support and, sometimes, consolation. But they misinterpret that role if they understand it as consisting of ‘spiritual interventions or therapies’, exclusively aimed at the alleviation of the patient’s suffering. The patient’s actual situation can be such that he would have to live in a world of illusion in order *not* to suffer. To the extent that grief, sadness and other negative emotional states are appropriate responses to the actual circumstances in which the patient finds himself, they are not to be considered ‘symptoms’ of a pathology, to be fighted at all costs.

In “An illustration: dignity therapy” section I will illustrate my criticism by discussing the famous ‘dignity therapy’, developed by Harvey Chochinov and his associates. In "Some major elements of suffering" section I will consider some important dimensions of suffering at the end of life in order both to further specify that criticism, but also to nuance it. In considering the facts as they are we should consider them from the personal perspective of the patient, even when that perspective as such is vulnerable to criticism. And when suffering reaches a peak where the patient is helpless in the face of an overwhelming force, we should no longer be scrupulous about selling him illusions.

Suffering, I will conclude ("Conclusion" section), is sometimes and to some extent a condition that requires to be respected. I do not dispute that the alleviation of suffering is the main aim of palliative care for the dying. But pursuing that aim should remain within the boundaries of a constraint of realism.

The direct object of my criticism is a certain literature, the new empiricial literature on the dimensions of suffering, not the general literature on palliative care, nor it’s practice. Although the views that I criticize are fairly common in practice as well, I am sure that many doctors and nurses try to find an equilibrium between relieving suffering and respecting suffering. My aim is to explain why this is the right attitude.

## Cassell on suffering

As we saw Cassell conceives of suffering as being distressed by circumstances understood as threats to one’s person. The term ‘distress’ obviously refers to a state of consciousness. If something happens that you consider undesirable, you only suffer from the event if you experience its occurrence or its effects with grief and sorrow. These are negative emotions, but their negative character is not enough for recognizing you to suffer. You should understand the event or it effects as endangering the core of your personhood.

This means that suffering is an intentional state, not merely a sensation with a certain tonality. In this respect it is similar to happiness.^3^ All intentional states have two aspects: the state itself and its object. If you believe, hope, fear or desire, there has to be *something* that you believe, hope for, fear or desire. This something can be unpacked as a proposition: you believe that something is the case, or you hope, fear or desire that it will be. Many such states involve cognitive claims: if that ‘something’ is not really the case, your belief is false; and if there is no reason at all to expect it to happen, your hope and your fear are inappropriate. (But not your desire: if your desire doesn’t fit the world, it is rather the world that is ‘at fault’^4^.) Suffering may involve a whole range of negative emotions: in addition to grief and sorrow, anxious concern, fear, anger, despair, frustration, bitterness, guilt, embarassment, shame. These emotions all have their own cognitive element: you are angry because you think that people are unfair to you, or Fate is, you are desperate because something that is extremely dear to you is irrevocably escaping you. But suffering, according to Cassell, has a cognitive aspect of its own: it implies the belief that your personhood is fundamentally threatened.

As Cassell explains in his topology of personhood, the threat may be directed at different aspects of your person.^5^ Most basically, you may consider your biological life to be at stake, and even if you don’t attribute any inherent value to being barely alive, it is a necessary condition of everything else that is valuable to you about your life. Your illness and its effects may also endanger you as the subject of the story of your life, by making it difficult or impossible for you to go on contributing to causes that gave meaning and coherence to that story. Perhaps even more fundamentally it may endanger you as a person having a life of her own to live at all, by invading your daily routines and corroding your intimate relationships. You may also feel that you are no longer your normal self in other social contacts, because you are losing the essential elements that determine your role and status in those contacts: the person you are, and the person you think you are, in the eyes of others. As a result your self-esteem may be endangered as well. In all these respects there is a growing disparity between what you are accustomed to do and to be and what you find yourself to be able to do and to be.

Suffering never consists of pain, dyspnea, nausea, fatigue or other physical symptoms alone. Pain, for example, is an element of suffering, not merely a cause, but in order to know, when someone is in pain, whether she is also suffering, we have to understand what the pain means to her. The pain in childbirth, to cite the standard-example, may be extreme, but it is rarely considered an element of suffering. Pain of the same severity would definitely be an element of suffering if it didn’t announce a new life but death. It is not only the case that the meaning of the pain determines to what extent you are suffering, it even determines the nature of the sensation itself: how painful the pain really is. If you learn that the pain you are feeling during an operation will be over in a minute, it is already less biting. On the other hand, fear and anxiety can intensify restlessness, vomiting, sleep disturbance and other physical symptoms.^6^

If we understand suffering in the way Cassell proposes, it can never be a good argument, in assessing someone’s suffering from a certain disease, to say that other people who are in exactly the same physiological condition, do not suffer, or do not suffer severely. For whether this condition can intelligibly be perceived by someone as endangering her integrity as a person, will depend on the person she is, her character, her life history and her values. An amputation may mean something completely different to you when you have been a dancer all your life than when you have been an arm-chair philosopher. In this sense suffering is a subjective matter.^7^

## Empirical work on suffering

The new literature on suffering, mainly focussing on the end-stage of life, aims to provide us with an organized list of elements of suffering, informing us for each element about its incidence, and sometimes also giving us scores for the average severity of the suffering on that particular dimension. Such lists are only meant to offer a checklist for understanding the specific nature of a person’s suffering, they are not supposed to be usable for calculating an overall score of the severity of that suffering. Usually the elements are grouped into four classes: physical symptoms, psychological, existential and interpersonal or social dimensions of suffering.^8^ This standard classification, however, is not very stable: the same or similar aspects are often differently classified.^9^

On closer look the so-called physical symptoms are all mental states. They are only different from other sensations and perceptions because of their relation to a more or less clear bodily location. If it itches, it itches somewhere. Coughing, vomiting and bodily disfigurements are forms of suffering because of the way these physical states and events are experienced. Some symptoms mentioned on the lists are clearly perceptions, for example smelling unpleasant, others seem to be mere sensations, for example itching.^10^

When Cassell wrote his paper, it was common for doctors to understand the very term ‘suffering’ to refer to pain. Although this is no longer true, many doctors still have a narrow focus on physical symptoms.^11^ Some studies have found that physical symptoms are the most important elements of suffering, in terms of incidence and of average severity.^12^ But according to most studies the non-physiological elements are more important. The second conclusion seems to be confirmed by the findings of studies of the reasons people have for requesting physician assistance to end their lives. Existential and social concerns are often more prominent on these lists than physical symptoms.^13^

The list of possible physical symptoms is very long,^14^ but the most prominent seem to be: fatigue, pain, breathlessness and nausea. Fatigue (including weakness, exhaustion, cachexia) seems generally to be the most common.^15^ An important finding is that even for patients who we may assume to receive the best possible palliative care, for example in a hospice, it is not uncommon that they rate the pain and other symptoms they experience as severe.^16^ On the non-physiological dimensions the variation in the relevant aspects is much greater. Understanding oneself to be a burden for others, for example, is very prominent on some lists but absent on others.^17^ But, if one goes through a substantial number of these research reports, a fairly coherent picture of the most important dimensions emerges. (See § 6) It is confirmed by the studies of the reasons for requesting physician-assisted death.

As I said, almost all these studies refer to Cassell, and one idea that they have all learned from him is that suffering cannot exhaustively be described in terms of physical symptoms. But in other respects they are not at all true to his insights. This is particularly true of the studies that do not only give lists of elements of suffering, but try to measure their importance. If, for example, a person is extremely tired, it will be difficult for her to engage in meaningful activities, to take care of her own needs or even to find a way of coping mentally with her condition. Fatigue makes the hard work of dying even harder. So when we ask her to tell us how important fatigue is as an element of her suffering, do we mean: fatigue as such, abstracting from these further effects? If that is what we mean, we do not really accept Cassell’s point that in order to determine the severity of a person’s suffering from fatigue we have to consider to what extent this physical symptom is understood as threatening. But if we are prepared to take these further effects into account, we are already measuring a psychological or existential dimension of suffering, not a mere physical symptom.^18^

Making listst of elements can only be the first step in understanding suffering. In order to really understand it we have to consider the interplay of these elements. If your pain is rated as an important element of suffering because it interferes with your activities, that effect may be reinforced by your sense that these activities have lost their point anyway. If you are already suffering from losses of meaning and of autonomy because of further losses of sight, hearing and mobility, that effect can be multiplied by fatigue and by fear. Hence we cannot assess the extent of a person’s suffering by considering the elements of suffering one by one, let alone by scoring their severity one by one.^19^ Holism is an essential characteristic of Cassell’s conception of suffering.

An additional problem of using measuring scales is the problem of interpersonal comparability. Perhaps the person who gives his suffering the highest possible score today will discover tomorrow that it can still become much worse. So how do we know that the same score indicates a comparable level of suffering?

A final common weakness of the existing studies is that they don’t recognize the dual nature of suffering as an intentional state. Some of the items on their lists only refer to the subjective side of the intentional state, others to such states identified by their object, or even only to the intentional objects as such. One study, for example, includes the following elements as ‘personal aspects of suffering’: feelings of guilt, of worthlessness, of loneliness, of hopelessness, feeling no longer the same person, feeling tired of life, feeling dependent on others, feeling loss of control, feeling being a nuisance, feeling not important to others.^20^ All these ‘feelings’ are identified by their object. Another study lists the following emotions as ‘psychological symptoms’: being afraid, worried or anxious, irritable, depressed, hopeless, sad, burden to others (sic), angry, lonely, embarrassed about oneself, guilty, abandoned, rejected.^21^ Most of these ‘feelings’ are described in purely subjective terms, but if we ask, what makes these people afraid, worried, angry, et cetera -referring by that question to the objects of those intentional states, not merely their causes-, the answers obviously are such things as: present or future dependence on the care of others, loneliness, being a burden to others, abandonment, rejection. This means that basically the same items may occur on the list within several classes, in particular as psychological and as social or existential elements.^22^

But the real problem is not only a matter of double counting or of an inaccurate system of classification. It goes much deeper.

## The constraint of realism

That basic problem is the following. If you focus on the subjective side of the intentional state and not on the intentional object, it becomes easy for you to treat all the elements of suffering in the same way as physical symptoms. The intentional objects are then only seen as accidental causes of suffering, the suffering itself is identified with the subjective state which consists of all the physical and emotional ‘symptoms’ taken together. Some of the earlier studies of ‘physical and psychological distress’ used only this simple dual classification.^23^ But even when ‘feelings’ are identified by their objects and classified as existential or social concerns, the understanding of suffering as a basically pathological state doesn’t change. Loss of faith is then simply one other item to be taken into account in determining one’s score on the ‘distress thermometer’, together with indigestion and constipation.^24^ The obvious aim of palliative care remains to reduce all symptoms, from whatever category, as much as possible. The checklists are supposed to enable us to concentrate our efforts on the relevant elements. They are instruments for diagnosis, ideally to be followed by treatment. We could call this a purely functional approach to suffering: if we come across any case of suffering, we have a reason to do something about it.

This approach fails to recognize the cognitive aspect of all emotions, including the negative ones: they are all more or less adequate responses to the situation the person finds herself in. The same is true of suffering as an overall response to that situation. To give one example: many studies seem to use the term ‘depression’, either as an equivalent of ‘sadness’, or as an indication of a high degree of sadness. This suggests that sadness as such is a pathological state that should always be attenuated as much as possible. But the essential characteristic of depression is being sad without loss, and when you are acutely aware of, for example, losing all options for meaningful activities and self-care, it would rather be pathological for you not to be sad. Sadness is by itself a mood, not a mood disorder, and hence not necessarily something to be avoided or minimized.

Even if you meet some other standard conditions of depression as listed in the DSM-V, that may also be something to be expected in the situation. Either your illness itself or its physical symptoms may already rob you of your sleep, but if reflection on your situation does so, that is not necessarily a sign of a psychiatric condition either. To recognize that negative emotions may be appropriate responses to reality could be called a realistic approach to suffering.^25^

Or consider fears for the future. Such fears may concern the further development of the illness with its accompanying symptoms, possible treatment outcomes, including side-effects, as well as death. Fears may also be directed to increasing social stigma or isolation. Such fears may be exaggerated, and they often are, but they may be also be fully warranted. In that case they should not be treated as the expression of an ‘anxiety adjustment disorder’. We should rather consider what we can still do to prevent them from being fulfilled.

But why shouldn’t we take a purely functional approach? Would that not be much better from the point of view of the patient? After all, if we succeed in reducing his negative feelings, however appropriate, it would result in a higher level of happiness and well-being for him, a higher quality of life. Wouldn’t it?

That remains to be seen. A purely functional attitude could easily become counter-productive. By now that has generally been acknowledged for the attempt, once usual, to keep the patient’s diagnosis and prognosis a secret for him. That can result in torturing uncertainty and make it impossible for the patient to make wise choices as regards the withdrawing of treatment and forms of palliative care with side-effects for his level of consciousness. Generally speaking, bringing the patient in an artificially euphoric state may rob him of his sense of urgency for doing what his situation calls for, arranging his affairs, taking leave, perhaps transmitting family traditions, and most importantly, coming to terms with the end of his life.

But whether this really is all that important, depends on another question: what is a good life, and hence a good death, for a person? Is it only determined by the way in which she experiences the world, disregarding whether this experience is consonant with reality (experientialism)? This is a fundamental philosophical question on which a large literature has appeared. Well-known is Robert Nozick’s thought experiment of the experience machine: neurologists have figured out a way to stimulate your brain to induce whatever pleasurable experiences you program to have. Once in the program you cannot distinguish between your experiences and those of real life. Would you prefer to be hooked on to he machine for the rest of your life, with only some interruptions giving you opportunities for reprogramming? 26

I cannot enter into a full discussion of this large issue, but I will appeal to what seems to me one conclusive argument against experientialism. Imagine a man who believes that he has a very good life: he is successful in his professional life and appreciated for his expertise by his colleagues, he has a loving partner, etcetera. But all this is untrue: his professional performance is bad, his colleagues ridicule him behind his back, his partner betrays him with a lover. Suppose that he continues in his false beliefs until his death. Has he had a good life?^27^ Thomas Nagel’s answer to the question is the following: we all agree that it would be very bad for this man to discover the truth. But how can it be bad for him to come to know the facts, if these facts are not bad for him in themselves?^28^ And by leaving him to live in his illusionary world, his colleagues and his partner bereave him of any opportunity to try to improve things. Only the truth can liberate us. It is therefore better to recognize the facts as they are with sadness than to live in blissful denial of them.

Some supporters of a purely functional approach don’t call this realism but rationalism, and suggest that it betrays a typical Western overrating of cognitive abilities. But that suggestion on the one hand neglects the fact that emotional states are characterized by their cognitive content as much as by their felt quality (‘what it is like to be’ sad, angry, shameful etc.). On the other hand the suggestion doesn’t take into account that our cognitive abilities, including the ability to respond with adequate emotions, are the main resources we have at our disposal for coping with the vicissitudes of life, including the consequences of illness.

I do not deny that the alleviation of suffering is the most important aim of palliative care. If fatigue, pain, breathlessness, nausea can be reduced, that will reduce suffering as well, and by itself, abstracting from further side-effects, be only highly beneficial to the patient. He is no longer in a state in which it matters that symptoms have a functional value of their own, that, for example, pain alerts to tissue damage and sickness helps to save energy. But as regards to the cognitive dimension of suffering, suffering as a response to a threatening situation, it should not be seen as merely consisting of such symptoms. Rather the adequacy of the response should be an object of reflection and of dialogue, with family and friends and, maybe, spiritual counselors. Nurses and doctors can to some extent participate in this dialogue, in particular when the patient’s way of coping with his condition is relevant to medical decisions, perhaps occasionally also outside the context of medical decision-making altogether, if such contacts are welcomed by the patient and are not considered to intrude into his private life. But when we talk with a fellow human being about his hopes and fears, the meaning of his life, his difficulties to accept his mortality or to preserve his self-respect, we are no longer involved in therapy.^29^

## An illustration: dignity therapy

Harvey Chochinov is one of the researchers of end-of-life suffering who is most true to the person-oriented and holistic character of Cassell’s programme.^30^ With his associates he has in particular done important research on loss of dignity as a dimension of suffering. In response to these findings they have developed a ‘psychotherapeutic intervention’ that they call ‘dignity therapy’. Although I do not know to what extent the therapy is actually being used in palliative care, it certainly has become quite famous.^31^

Actually the aim of the intervention is much broader than to restore a sense of dignity and self-worth, it is also concerned with losses of purpose and meaning, the loss of hope, and even with the loss of the ability to cope with the effects of illness. Patients who consent to participate are offered the opportunity to address issues that matter most to them or speak to things that they would most want to be remembered for as death draws near. An edited transcript of these sessions is returned to the patients for them to share with individuals of their choosing. The discussion is structured by a series of interview questions, such as the following:

- Tell me a little about your life history; particularly the parts that you either remember most or think are the most important? When did you feel most alive?
- What are your most important accomplishments, and what do you feel most proud of?
- What are your hopes and dreams for your loved ones?

My problems begin when an instrument like this is called a ‘psychotherapy’ or a ‘non-pharmacological intervention’.^32^ The very terminology shows that, although the suffering individual is supposedly addressed as a person, he really is only being treated as a patient, and his ‘feeling undignified’ as a symptom to be relieved. The method used to achieve this is to represent the patient’s life in the rosiest light possible, by focussing on the positive aspects of that life. Surely, it may be nice for you to be enabled to pass on the resulting document to your loved ones. More than 90% of the patients who consent to being interviewed welcome the opportunity. But perhaps your children would have preferred to receive a more honest assessment of your merits instead of this piece of self-advertising, maybe even a request for forgiveness for the ways in which you have made life more difficult for them, by your indifference or your relentless ambition. An instrument to pretty up your self-image is not necessarily the best you could give your children to remember you by.

It is important to strike the right balance here. I do not dispute that many people in the end stage of their illness are in a state of despondence that causes them to have a unduly negative image of their present and past selves. It will help them to be consoled, and counting their blessings may be a proper form of consolation.^33^ My point, however, is that not every negative assessment is an unduly negative one. This should be taken into account even in consoling patients. (Such realistic consolations may also be the more effective ones, but that is not my point).

The ‘dignity therapy’ turns out to be a kind of experience machine *light*, you could indeed imagine the same result to be achievable by the administration of a drug. In many respects, it seems to me, opium is to be preferred.^34^

## Some major elements of suffering

In order to flesh out the realistic approach we would have to consider in detail what it means in respect to some components of suffering, described in terms of their intentional object, that on the evidence of the empirical studies seem to belong to the most important ones, both in terms of incidence and of severity. I would, for example, have to discuss loss of hope as a dimension of suffering, and in particular the functional and inherent values and disvalues of false hope.^35^ My conclusion would be that there is often a better alternative to false hope than despair: acceptance. Actually at the moment of death most patients are at least resigned, if not reconciled to the fact.^36^ However, reflection and dialogue are required in order to achieve that stage, and when false hope is maintained too long, the remaining time to go through that process may become too short.

I would have to discuss loss of meaning and purpose, and in particular whether the personal value of a person’s life is enhanced by success in whatever she has set out to achieve in that life, even when this achievement is trivial or positively pernicious (Cf. footnote 23).

I would have to consider the common assumption of suicidologists that it is always a pathological symptom to believe that you are a burden to others.^37^ As a matter of fact the belief may be fully warranted and you may have compelling moral reasons to take the fact into account.^38^ The same point can be made about fears of future suffering. To reassure the patient about the possibilities of symptom management is important, but it should not come down to making false promises.^39^

In the remainder of this section, however, I will focus on two other dimensions of suffering, because considering them will enable me to point to some important limitations of the realistic approach I have advocated in this paper. These elements are often confused with each other, because both may be meant by terms such as ‘loss of autonomy’ or ‘loss of control’.^40^ They should, however, be clearly distinguished.

For many patients it is a major element of their suffering that they no longer can take care of themselves, even for the most basic elements of self-care. They may be bedridden, perhaps incontinent, they may not even be able on their own to take food or drink, or to remove a fly that tickles their nose.

When this is a dominant element of a patient’s suffering, we might want to argue with him. Look, we might want to say, human beings are always vulnerable, every moment they may be the victim of an accident that makes them dependent on the compassion and care of others, good Samaritans or professionals. As the Stoics knew already, it is a matter of true independence to be able to cope with that condition, and with the cutbacks on privacy it implies, even to the point of being able to be thankful to your nurse, or to correct her if she is unduly paternalistic.^41^

It may occasionally make sense, in particular for an intimate, to point this out. But I do not think that it is always a requirement of realism to do so. If a person has lived in an illusion of self-sufficiency all his life, and believes that it is abnormal or even humiliating to be dependent on the care of others, that is by now a relevant fact about his identity as a person. We would not make his life a better life (for him) if we somehow succeeded in preventing him to live by those values. We need not commit a relativistic fallacy—“it is good for him if he thinks it is”—to respect this element of his identity, in particular when we only enter his life in a professional role, as a doctor or a nurse. It would be disrespectful to try to educate him now, and it might add to his suffering, which, following Cassell, we should assess from his perspective.

The other major element of suffering that can be referred to by the term ‘loss of autonomy or control’, is the loss of coping resilience. That this is not identical to the loss of the ability of self-care, is shown by the fact that thát particular loss is one of the conditions that some people succeed in coping with. Suffering occurs when we understand our condition as threatening core aspects of our personhood, but in many cases people are able, with a lot of effort and almost always with the support of others, to mentally cope with that threat, to keep standing on their legs and walking, however insecurely. You can come to accept the fact that you will die soon, that you will no longer be able to contribute to causes that are dear to you, that you can no longer take care of yourself, and even that you are in a pitiful condition to behold for others. But in other cases people do not succeed in this task. If that happens to you, you can be said to succumb to the attack on the integrity of your person. That shows that your coping resilience is itself the most central aspect of your personhood. There is an (inverse) conceptual relation between the perception of a threat to your personhood and your awareness of resources to cope with that threat, including hope, a sense of meaning, of self-worth, or of connectedness.^42^

Coping resilience may itself be very much assailed by physical and mental symptoms such as fatigue or frailty, physical and mental exhaustion, nervousness, confusion, concentration problems. For such reasons the patient may not be able to think clearly. If your condition is one that you haven’t been in before, if the task of handling it weighs heavily, and your coping abilities are curtailed, your autonomy will even more than usually be in need of being ‘scaffolded’ by the support of others.^43^ But this support will not aways be available and sufficient.

Some ways in which people try to adjust to their situation, sometimes resulting from obsessional personality traits, are inept, even counterproductive ones. This may be true of emotional states like frustration and anger, insistence on improper forms of care or phobic avoidance of other forms. In such cases the functional and the realistic approach point in the same direction. Denial is also a very common coping strategy that in the end may lead to more rather than less suffering.

Precisely because coping resilience is an essential element of intact personhood, it is the worst kind of suffering if you are overwhelmed by grief, despair, anxiety, anger and guilt.^44^ If that happens it would be pointless to insist on realism, to go on requiring you to take an appropriate attitude even to that situation. If nothing else helps, mercy may require to sedate you. Some guidelines for Palliative Sedation explicitly allow this, attributing to you (characteristically) a ‘refractory existential symptom’.^45^ Yes, sedation for these reasons may be considered the use of an experience machine, at least as long as sedation is only mild. But under these circumstances that use may be entirely warranted.^46^

## Conclusion

What is basically at stake in this discussion is a normative conception of dying well. The functional approach rests on the ideal of a patient who is enabled to die in peace because both his physical symptoms and all other elements of his suffering are under full medical control. All deviations from this ideal are seen as defects, to be repaired as far as possible by therapy.^47^ Family support and spiritual care are both fully in the service of this aim. Every practititoner knows of course that this ideal is not always attainable, that not all suffering can be taken away. My point is that, even if that end could be achieved, we shouldn’t aim for it. Some aspects of suffering are beyond the legitimate scope of palliative care, even if such care is understood, as it should be, in a personalized, relational and holistic way.

For many patients their main concern in this phase is to round off the story of their life in a fitting way. It can only be fitting if it continues to be sensitive to the world in which they lives as it is, even if this means recognizing highly disquieting facts for what they are. It is true that there is often a spectrum of responses that can be seen as appropriate to adverse circumstances, and it is also often hard to know what is an appropriate, let alone the most appropriate response. Therefore in the search of an appropriate attitude there is not only room for reflection and discussion but also for support and consolation. But these should, with the exceptions that I noted in the last section, remain within the constraints of realism.

At this stage it is almost always helpful to soothe persisting physical symptoms of the terminal illness because they can no longer serve any beneficial function. But we shouldn’t conceive of all elements of suffering on the model of distressing physical symptoms, as phenomena that it is always in the interest of the patient to get rid of, whether by pills or by talk.^48^

Paradoxical though it may sound, suffering is a condition that, to some extent, requires to be respected as an integral part of dying well.

## Footnotes

1 Cassell (1982). Included in Cassell (1991).

2 Cassell (1982, p. 640, 1991, p. 33, 1999, p. 531).

3 On every understanding of happiness, even a hedonist one, cf. Feldman (2010, chs. 6–7).

4 Cf. Anscombe (1963, 56) on what has come to be called the direction-of-fit criterion for classifying intentional states (and speech acts).

5 In this paragraph I do not aim to summarize Cassell’s topology but reorder it in anticipation of my discussion in § 6.

6 Wall and Melzack (1999, ch. 1).

7 Cassell, however, seems to understand it as a subjective matter in a stronger sense: as something that is only accessible to the subject and can be established by her with final authority. Even people who know her person, her biography and her views and can take into account her behaviour and body language, have no independent access. For criticism of that view see Wijsbek (2012).

8 Cherny et al. (1994) seem to have introduced this fourfold classification.

9 For example, anxiety as a physical or as a psychological symptom. For other examples see Footnotes 20, 21 and 22.

10 It is disputed whether pain is a mere sensation normally caused by tissue damage or a perception of injury, see the introduction to Aydede (2006), or the same author’s entry ‘Pain’ in the Stanford Encyclopedia of Philosophy.

11 As a substantial number of Dutch doctors still do in assessing ‘unbearable suffering’ in the case of a request for euthanasia (Van Tol et al. 2010, 2011, 130–134; Pasman et al. 2009).

12 Benedict (1989), Kuupelomäki and Lauri (1998), Wilson et al. (2004), Wilson et al. (2007). Krikorian et al. (2012, p. 800), refer to a Spanish study by Maté, reporting that most caregivers attribute a peacful death to symptomatic control, most family members to psychosocial matters.

13 Van der Wal et al. (2005, 51), Pearlman et al. (2005), Ganzini et al. (2009), Pasman et al. (2009), Dees et al. (2011), Oregon Public Health Division (2014), Washington Death with Dignity Act Report (2014), cf. Dees et al. (2010) for a review of relevant studies. Exceptions include Meier et al. (1998), Wilson et al. (2007), Deliëns et al. (2011, chs 19–20). Note that we cannot assume that all patients who request physician assistance suffer severely.

14 The most detailed list, in Ruijs et al. (2009), contains 37 items, but some of these (feeling tense, depressed, anxious) are clearly misclassified.

15 Except for ‘general malaise’ or ‘general discomfort’ in a number of studies, e.g. Morita a.o. (2004). Kwon et al. (2006) found that pain was the most common and severe symptom, but fatigue the most interfering in patients’ lives. According to Oi-Ling et al. (2005) caregivers and physicians agreed with patients on the distress involved in most symptoms, but underrated fatigue and cachexia.

16 According to Baines and Norlander (2000), for example, 26% of hospice patients reported moderate, 24% severe pain in the last 24 h.

17 It is the most important non-physiological dimension of suffering according to Wilson et al. (2004), cf. Singer et al. (1999), but is not mentioned by Oi-Ling et al. (2005), or by Lloyd-Williams et al. (2008). McPherson et al. (2007) argue that it is an element of suffering that is systematically underrated by professional and informal caregivers.

18 Yun et al. (2006) differentiate between the incidence and severity of the symptom and its interference in the life of the patient (general activity, mood, work, relation, walking and enjoyment). The model of dignity developed by Van Gennip et al. (2013), distinguishes between illness-related conditions (physical symptoms, functional/cognitive ability, appearance) and their effects on ‘personal dignity’.

19 In various studies, e.g. Benedict (1989), Byock and Merriman (1998), Holland (1999/2014) (the ‘distress thermometer’), Schulz et al. (2010), Ruijs et al. (2012), subjects have been asked to give a separate overall score of their suffering. It is then unclear how this overall score is supposed to be related to the scores on individual aspects. A mysterious finding of Ruijs et al. (2012), for example, is that the number of people with an overall score of ‘serious or very serious suffering’ was lower than the number with the same score on some specific aspects. A very different measure of suffering is provided by the Pictorial Representation of Illness and Self-Measure (PRISM), developed by Büchi et al. (2002). Subjects are asked to put a red disk, representing their illness, on a yellow disk, representing their ‘Selves’. Suffering is measured by the distance between the centers of the disks: the Self-Illness Separation (SIS). As the authors note, this measure can be understood to correspond to Cassell’s conception of suffering.

20 Ruijs et al. (2009). Chochinov et al. (2009) mention the following ‘feelings’ among 25 aspects of distress in the terminally ill: feeling no longer who I was, feeling of not having control, feeling uncertain, feeling anxious, feeling of reduced privacy, feeling aburden to others, feeling how your look has changed, feeling depressed, feeling of unfinished business, feeling life no longer has meaning or purpose, bot feeling worthwhile or valued, feeling not have made meaningful contribution, not feeling able to mentally fight illness, not feeling supported by health care providers or by friends and family. Ruijs et al. (2014) observes the overlap between categories of suffering but fails to explain it.

21 Schulz et al. (2010). Cf. Kuupelomäki and Lauri (1998): anxiety, depression, feelings of anger and guilt, aggressiveness, irritability, nervousness, and various fears and worries; de Haes et al. (1990): irritability, worrying, depressed mood, nervousness, desperate feelings about the future, tension, anxiety and concentration problems.

22 Although Schulz et al. (2010) combined measuring instruments for the domains of physical symptoms, psychological distress and existential or spiritual suffering in order “to compile a comprehensive set of non-overlapping items”.

23 E.g. the Symptom Assessment Scale, Bruera et al. (1991), that is still widely used, cf. Richardson and Jones (2009).

24 Holland (1999/2014). Cf. the measurement scales for spiritual suffering at the end of life, developed by Peterman et al. (2002), and Steinhauser et al. (2006).

25 Kissane (2012) uses the terms ‘adaptive adjustment’ and ‘maldaptive response’ themselves in a purely functional way, for positive, resp. negative feelings and social environments. ‘Fear of being a burden’, for example, is then always a ‘maladaptive response’, whether that fear is warranted or not. ‘Fulfilled life, with accomplishments and legacy’ is then always an ‘adaptive adjustment’, even for someone like Ivan Ilyich. Kissane et al. (2001), cf. Kissane et al. (2004) even argues for the recognition of a ‘demoralisation syndrome’ as a new psychiatric diagnosis, characterised by loss of meaning, dysphoria, disheartenment, helplessness and a sense of failure. Slavney (1999) objected that demoralization is not a psychiatric disorder at all, but a normal response to adversity. Chapman and Gavrin (1999) consider stress responses to be possibly beneficial when they are short-term responses to a negative stressor, because they prepare the subject for an emergency reaction, but always maladaptive when the stressor persists.

26 Nozick (1971, 1974, 42–45, 1989, 104–108). Although suggestive, the question is less than fully compelling as an argument against experientialism because answers are sensitive to framing effects, and positive answers are not unknown.

27 Would Ivan Ilych have had a good life if he had suddenly died from a heart attack a day before the accident that lead to his terrible death?

28 Nagel (1970).

29 For similarly motivated reservations about palliative sedation (taking away the patient’s ability to deal practically and emotionally with his condition in an appropriate way) see Jansen and Sulmasy (2002), and Battin (2013), cf. my comments in….. Similar objections have also been made to pharmacological mood enhancement: “suffering from grief at the loss of a loved one is not the kind of… negative emotion one should want to ‘get rid of’ by means of a mood enhancer”, Schermer (2014), and cf. Olsen (2006).

30 See in particular Chochinov (2006), Chochinov et al. (2002, 2009).

31 Chochinov et al. (2005). Cf. Chochinov et al. (2011), reporting beneficial effects on patients’ level of well-being.

32 Similarly, some of the procedures, recommended by Chochinov (2006, 95ff). should be seen as requirements of humanity or professionality and not as ‘therapeutic interventions’ at all, for example ‘ask permission to examine patients’ or ‘treat the patient as worthy of honour, esteem and respect’.

33 Cf. the story of Jacques in Chochinov (2003).

34 Similar comments could be made to Wendy Duggleby’s Living With Hope Program, Duggleby et al. (2007).

35 For another recent purely functional approach to hope, see Olsman et al. (2014). Interesting attempts to balance functional and realistic concerns have been made by Ruddick (1999), Musschenga (2015).

36 Mack et al. (2008).

37 See Den Hartogh (2016a) for further comments and references.

38 Den Hartogh unpubl.

39 One example of a functional approach to fear: Karlsson et al. (2012).

40 This is the most important reason for a request for physician assistance to end one’s life in Oregon and Washington, Oregon Public Health Division (2014), Washington Death with Dignity Act Report (2014).

41 Cf. Boer (2012). Many Dutch doctors have some such uneasiness when aversion to being dependent on care is the patient’s main reason for requesting euthanasia, Van Tol et al. (2011, 132).

42 Krikorian et al. (2012). The PRISM-measure of suffering (see Footnote 14) was orginally developed as a measure for coping ability.

43 Using a term from Joel Anderson, cf. Anderson and Honneth (2005), and MacKenzie (2008).

44 See characteristic statements of people with a low SIS in Büchi et al. (2002): “My illness is always there, it will never go away…it’s all my life, it’s like the end of the world”.

45 Including KNMG (2009) and Zorgnet Vlaanderen (2012). For discussion see Den Hartogh (2016b).

46 Cf. Wijsbek (1999) about the circumstances in which it would be a good idea to hook on to Nozick’s experience-machine.

47 See also the Missoula-VITAS Quality of life index for patients with terminal illsness. (Byock and Merriman 1998, 243–244) The underlying *ars moriendi* is described in Byock (1996).

48 Cf. the WHO definition of palliative care: “an approach that improves the quality of life of individuals and their families facing the problems associated with lifethreatening illness, through the prevention and relief of suffering by means of early identification and impeccable assessment and treatment of pain and other problems, physical, psychosocial and spiritual.” (see website WHO).
